# Supplementary material for: Magnesium oxide-water compounds at megabar pressure and implications on planetary interiors
Source: Nat Commun. 2023 Mar 1;14:1165. doi: 10.1038/s41467-023-36802-8 (PMC9977943; doi:10.1038/s41467-023-36802-8)
Supplement: Supplementary file 1 — Supplementary Information [file 41467_2023_36802_MOESM1_ESM.pdf]

Supplemental Information for “Magnesium Oxide-Water compounds at Megabar Pressure and implications on planetary interiors”

Shuning Pan,[1,*] Tianheng Huang,[1,*] Allona Vazan,[2] Zhixin Liang,[1] Cong Liu,[1] Junjie Wang,[1] Chris J. Pickard,[3,4] Hui-Tian Wang,[1] Dingyu Xing,[1] and Jian Sun[1,†]

[1] National Laboratory of Solid State Microstructures, School of Physics and Collaborative Innovation Center of Advanced Microstructures, Nanjing University, Nanjing 210093, China

[2]Astrophysics Research Center of the Open University (ARCO), The Open University of Israel, 4353701 Raanana, Israel

[3] Theory of Condensed Matter Group, Cavendish Laboratory, J. J. Thomson Avenue, Cambridge CB3 0HE, United Kingdom

[4]Advanced Institute for Materials Research, Tohoku University 2-1-1 Katahira, Aoba, Sendai, 980-8577, Japan

[*] Shuning Pan and Tianheng Huang contributed equally to this work.
[†] Corresponding author. jiansun@nju.edu.cn

Definition of superionic phase. There are different quantifiable definitions of 'superionic phase'. In simulation works, superionic states are usually characterized by a finite positive diffusion rate of mobile atoms (H atoms in our case), while other atoms (Mg and O atoms in our case) vibrate at their equilibrium positions. If the diffusion rate of mobile atoms is larger than some cutoff value D, the state is defined as superionic. For example, in a paper about superionic BCC ice[1], the superionic regime is defined by $D_O = 0$ and $D_H > 0$ (D_O and D_H are diffusion coefficients of O and H atoms). In another work about superionic ice[2], the superionic transition temperatures are defined by $D_H = 10^{-8}$ m^2/s. In a recent work about superionic helium–water compounds[3], the authors use a cutoff around $D = 10^{-9}$ m^2/s ($D_H = 2.1\times10^{-9}$ m^2/s and $D_{He} = 3.3\times10^{-9}$ m^2/s are defined are as 'superionic' in their work).

In this work, we calculate the diffusion coefficients from the slope of mean squared displacement (MSD): $D = \text{MSD}/6t$, and define the superionic regime as $D_H > 10^{-9}$ m^2/s. Here are some diffusion rates from our simulations: at 600 GPa and 6000 K, $D_H = 1.11\times10^{-8}$ m^2/s in $Mg_2O_3H_2$. $D_H = 9.30\times10^{-8}$ m^2/s in MgO_3H_4, $D_H = 1.37\times10^{-7}$ m^2/s in MgO_4H_6. More data can be found in Table S2.

Electronic conductivity. The electronic conductivity of MgO-H_2O compounds is lower than pure H_2O. Here are reasons: we calculated protonic conductivity by Nernst–Einstein equation ($\sigma = DNq^2/k_BT$). In the equation, q is the carrier electric charge, D is the carrier diffusion coefficient, N is the carrier density, and T is temperature. Superionic phases of H_2O and MgO-H_2O compounds have the same charge carriers (protons), so q has no major effect on their protonic conductivity. The temperature condition of superionic H_2O conductivity data[4] is around 4000~6000 K. Our calculations cover this temperature range, so temperature does not cause the difference.

The carrier densities of MgO-H_2O compounds are lower than H_2O. At 400 GPa, the carrier density is 0.303/Å^3 in *Pbcm* phase[5] of H_2O, 0.079/Å^3 in $Mg_2O_3H_2$, 0.207/Å^3 in MgO_4H_6 (0.178/Å^3 in MgO_3H_4, but it is not stable at 400 GPa). Among them, H_2O has the highest carrier density, which is in favor of proton conductivity.

The proton diffusion rates of MgO-H_2O compounds are also lower than H_2O. At 3000 K, the proton diffusion rate is around 7×10^{-8} m^2/s in H_2O[2], which is about three times the value of MgO_4H_6 and MgO_3H_4, 20 times the value of $Mg_2O_3H_2$. This can be explained by the existence of Mg-O polyhedral, which hinders the proton diffusion. Similar results can be found in MgO-SiO_2-H_2O system[6] and SiO_2-H_2O system[7], where the proton diffusion rates are also lower than in pure H_2O.

Supplemental Figures and Tables

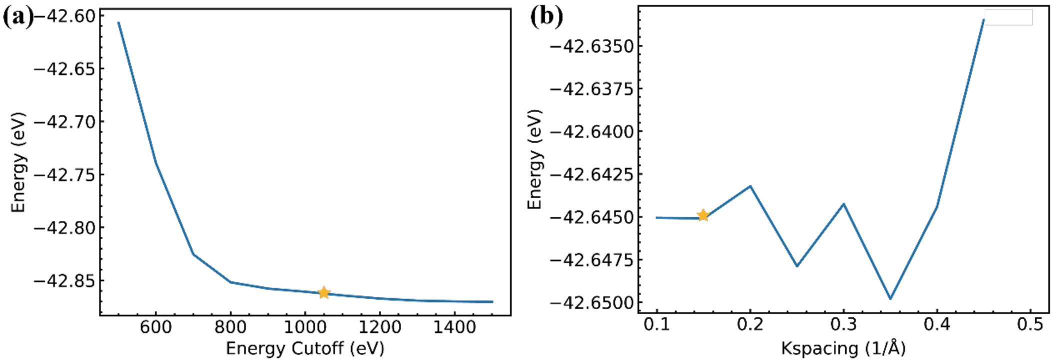

Figure S1 | Convergence test of DFT parameters. (a) Kinetic energy cutoff (b) Space between k points. The yellow star marks the value we used.

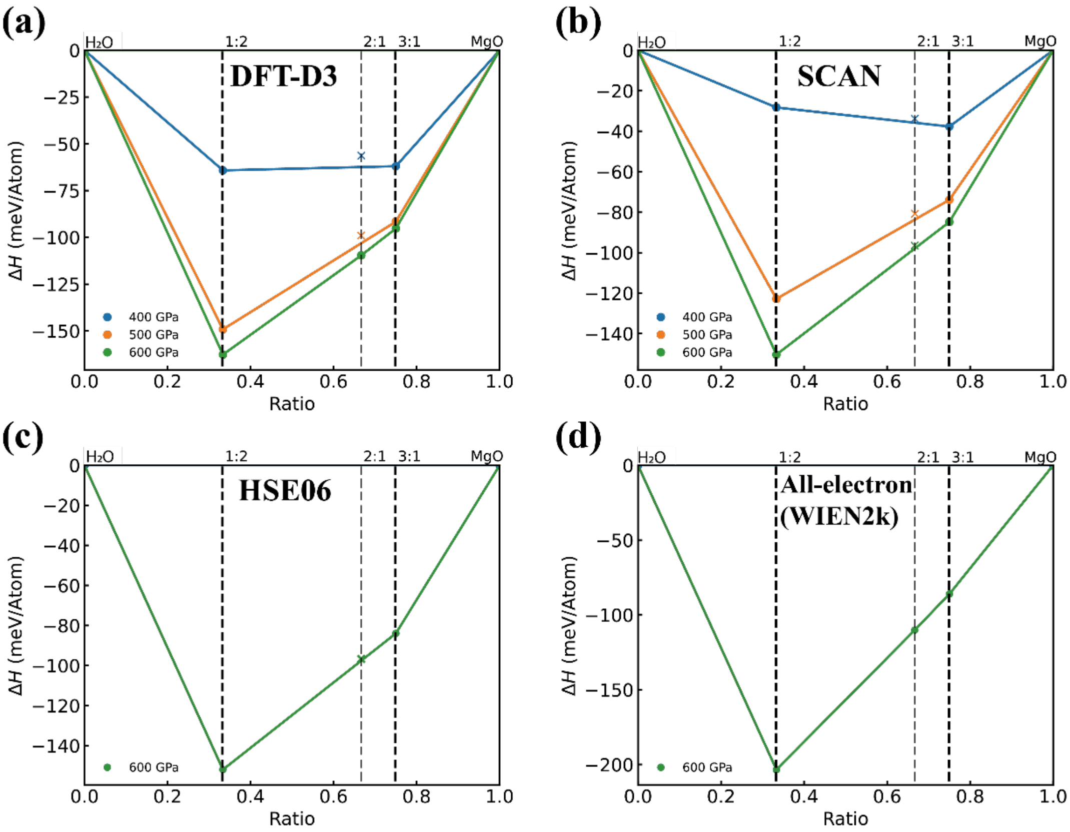

Figure S2 | Convex hull at 600 GPa, calculated by different methods. (a) vdW correction (DFT-D3) (b) SCAN (Strongly constrained and appropriately normed) functional (3) HSE06 functional (4) All-electron method by WIEN2k. We only calculate one pressure point in (3)(4) because the calculation is expensive.

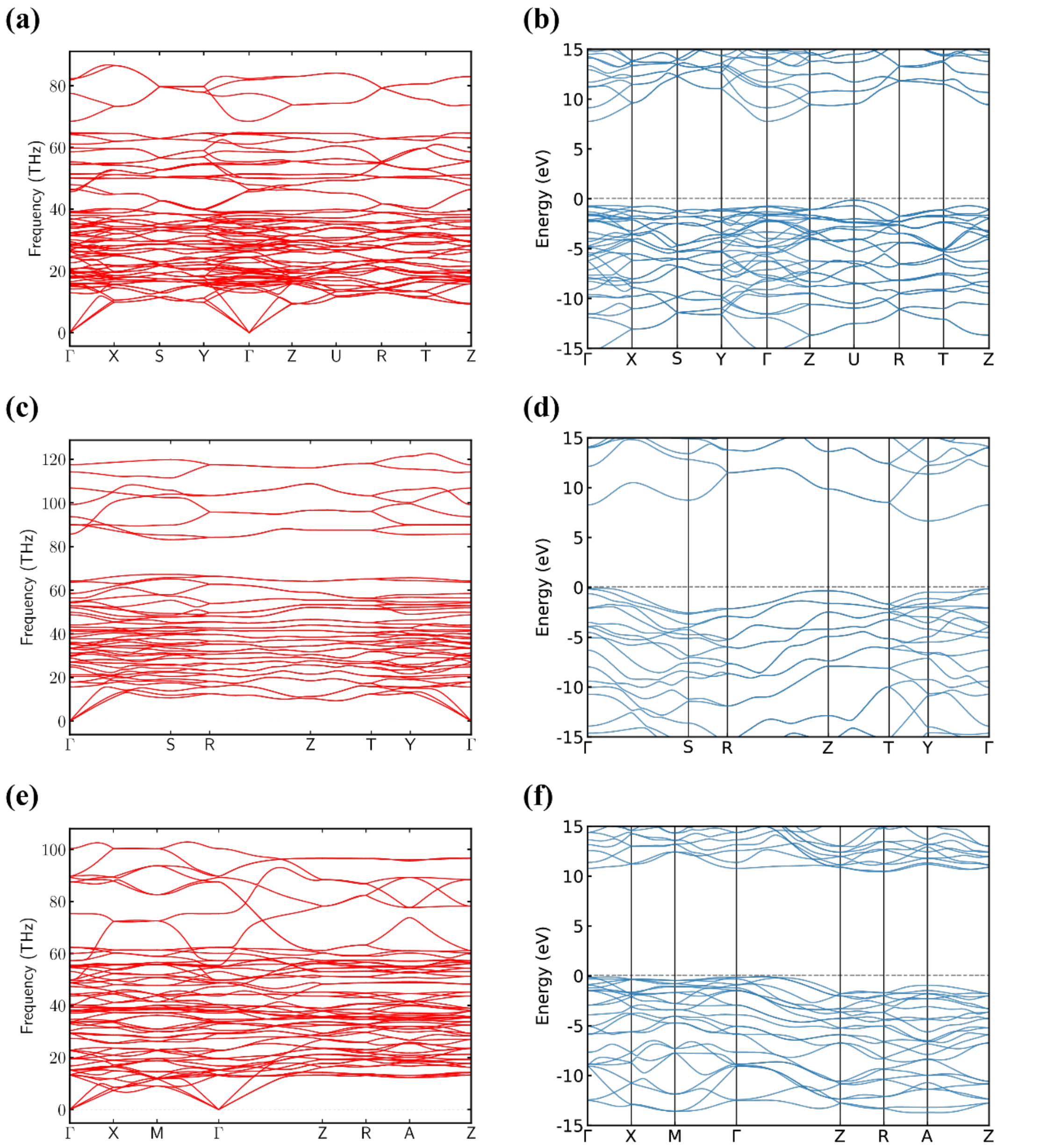

Figure S3 | Phonon band and electron energy band of MgO-H_2O compounds. (a-b) $Mg_2O_3H_2$ at 400 GPa. (c-d) MgO_3H_4 at 700 GPa. (e-f) MgO_4H_6 at 300 GPa.

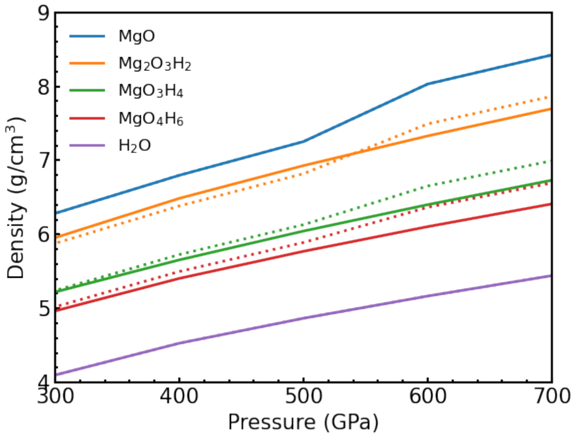

Figure S4 | The pressure-density relationship of MgO-H_2O compounds. The dotted line in this figure are calculated with Additive Volume Law.

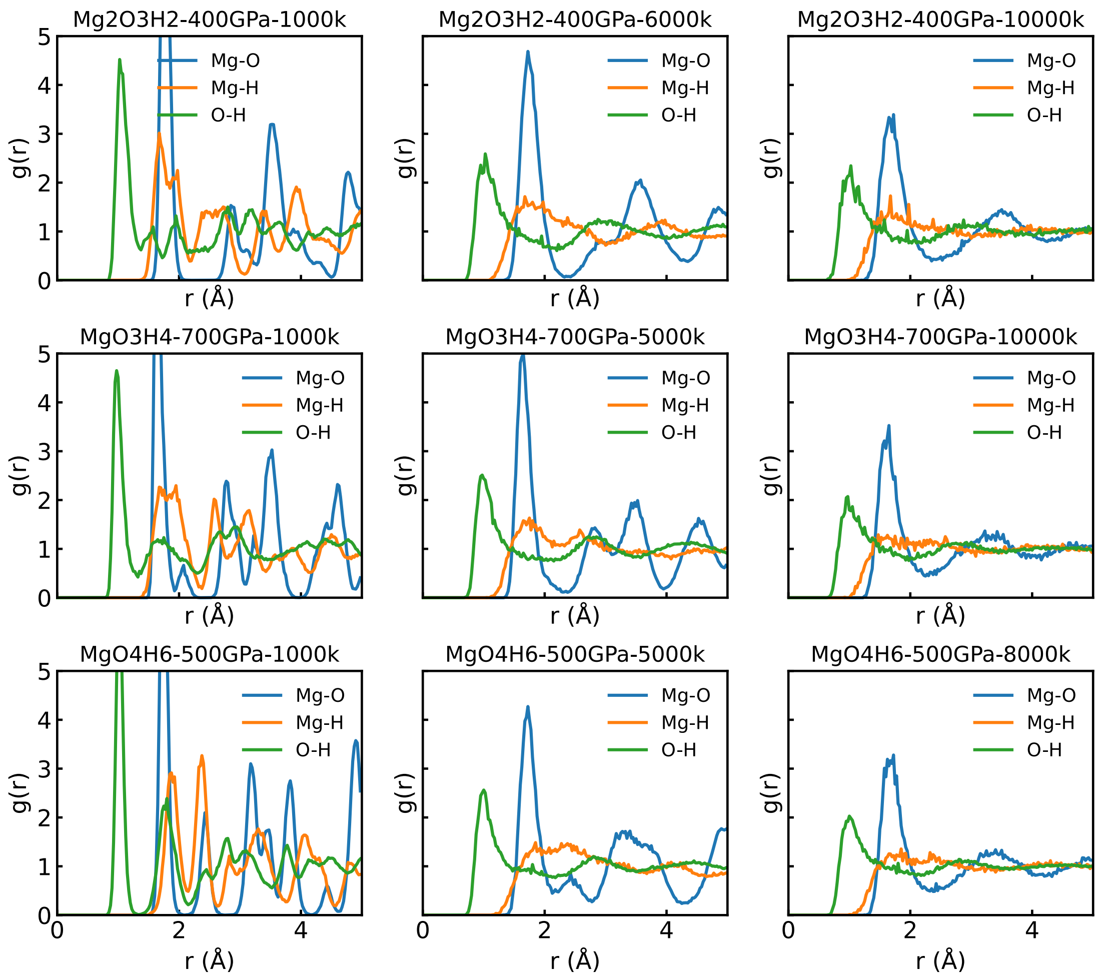

Figure S5 | The radius distribution function (RDF) of MgO-H_2O compounds at different temperature.

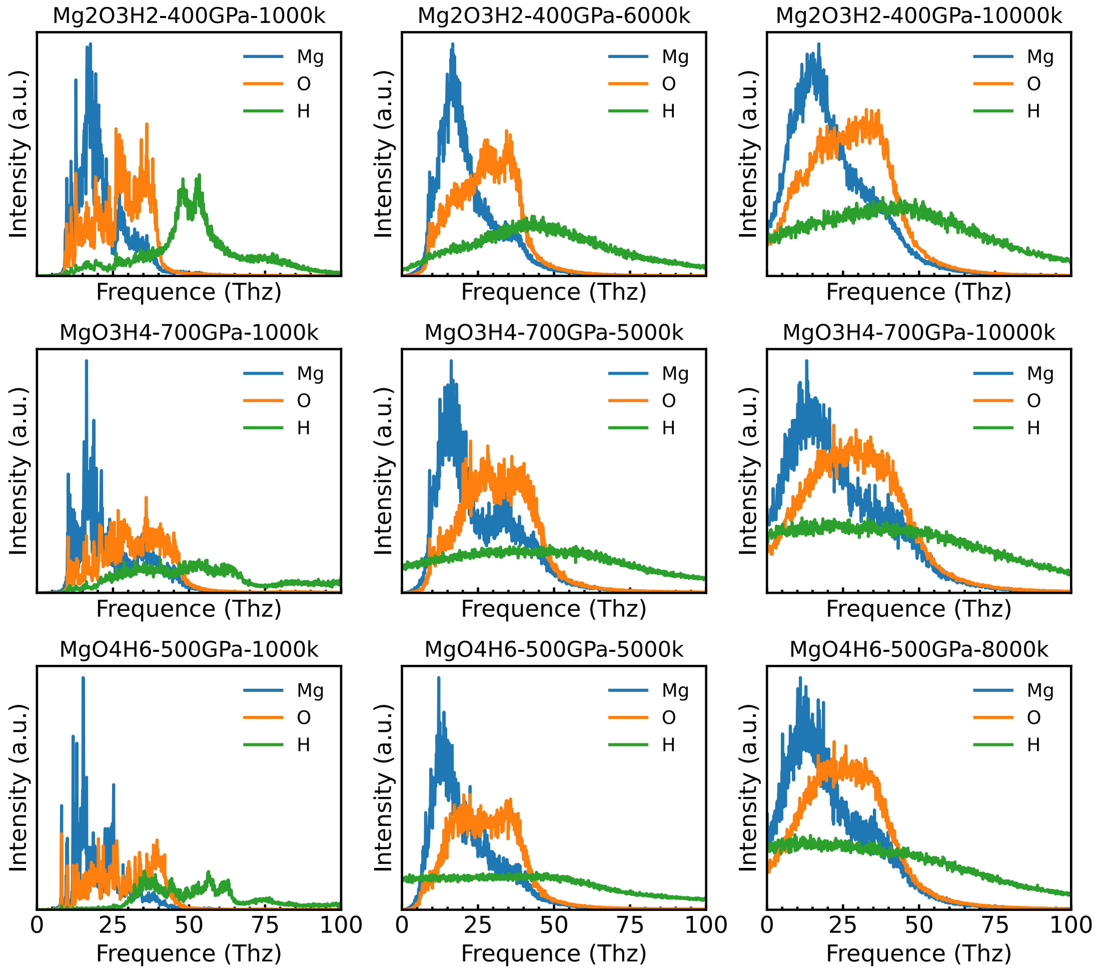

Figure S6 | The vibrational density of states (VDOS) of MgO-H_2O compounds at different temperature.

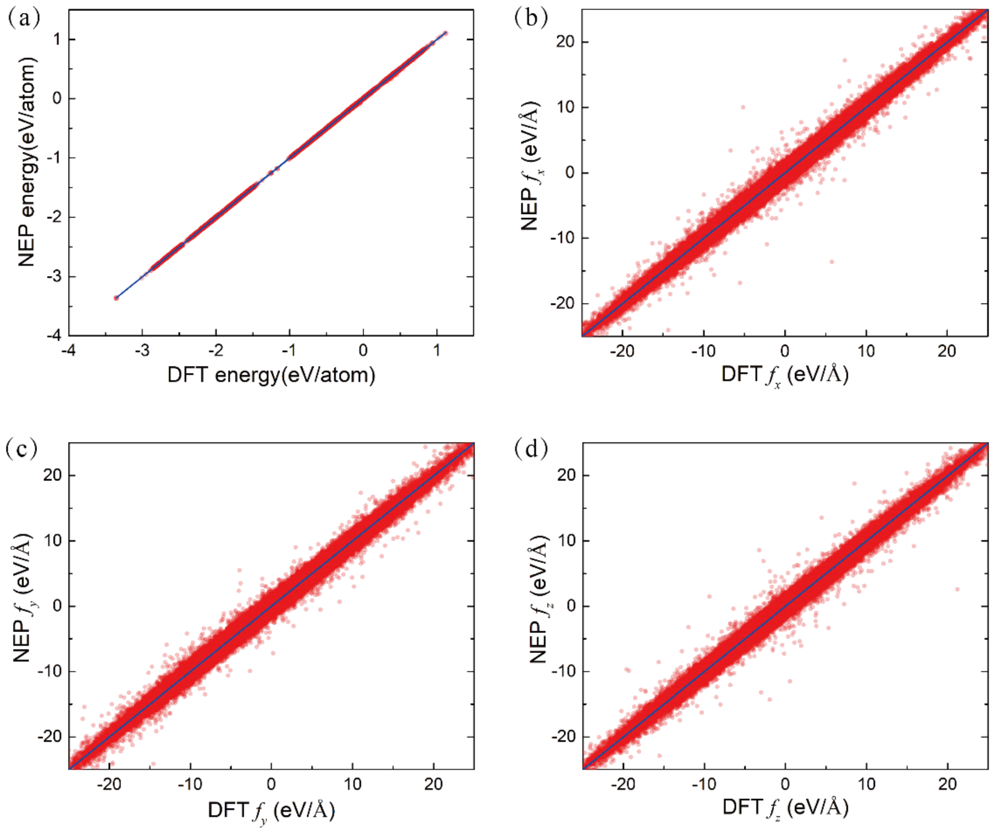

Figure S7 | The atomic energy (a) and the forces (b-d) calculated using the machine-learning neural network potential compared with the results from DFT calculations.

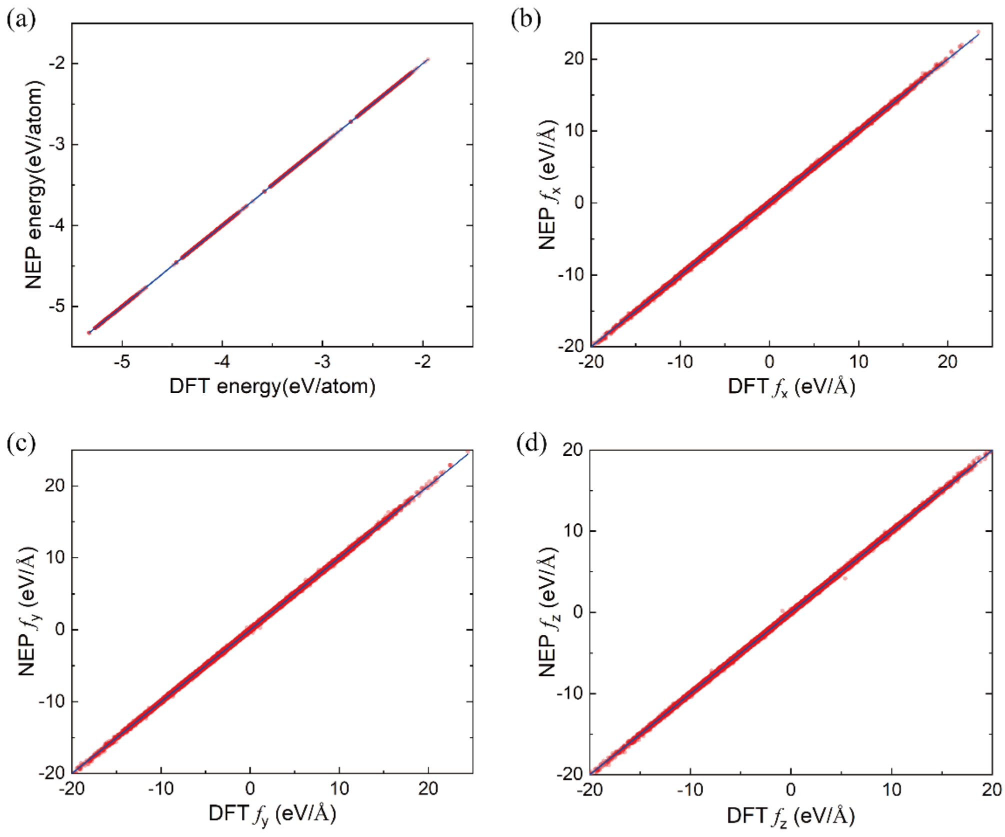

Fig. S8 | The atomic energy (a) and the forces (b-d) of the B1 phase MgO calculated at around 150 GPa using the machine-learning neural network potential compared with the results from DFT calculations.

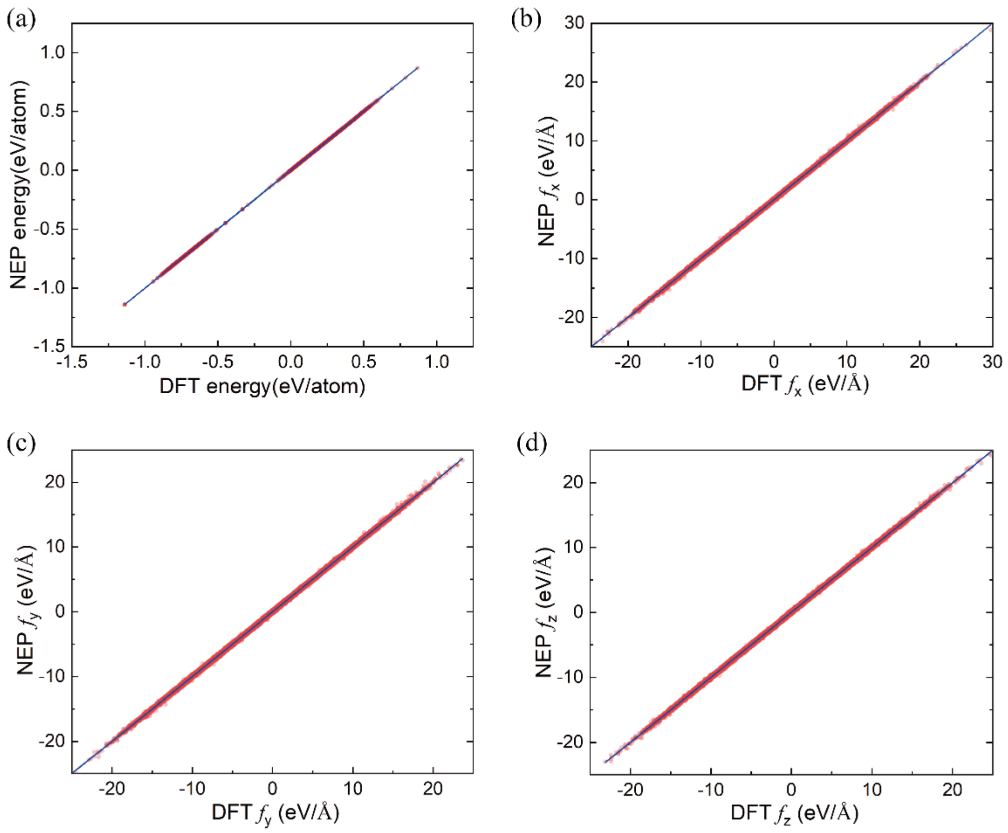

Fig. S9 | The atomic energy (a) and the forces (b-d) of the B2 phase MgO calculated at 600 GPa using the machine-learning neural network potential compared with the results from DFT calculations.

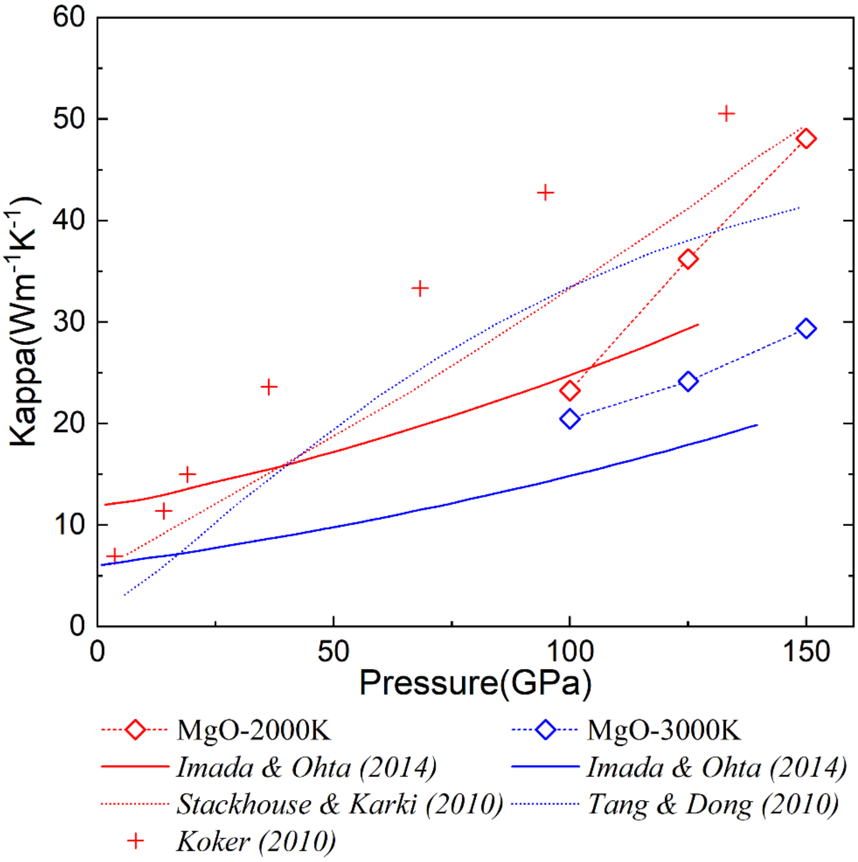

Fig. S10 | The thermal conductivities of the B1 phase MgO in the pressure range from 100 to 150 GPa, comparing with both experimental and computational results from previous work[8–11]. The solid lines represent the results of experiments and the dashed lines represent the results of theoretical calculations. The red and blue lines represent the results at 2000 and 3000 K, respectively.

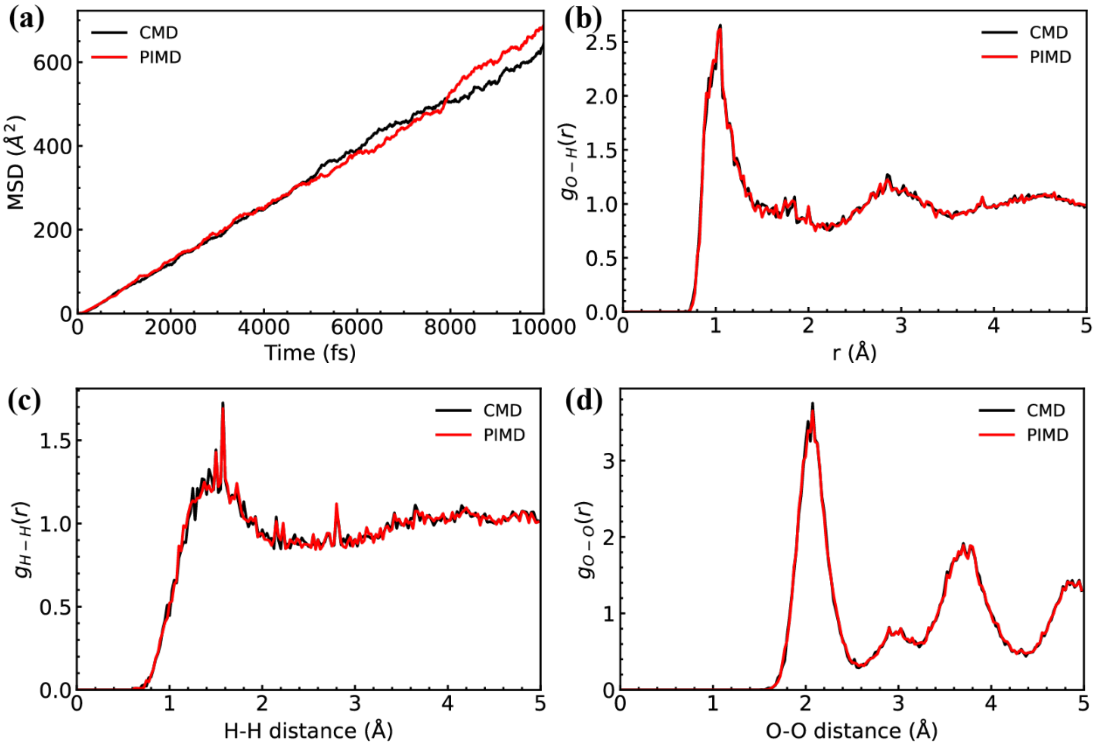

Figure S11 | We use path integral molecular dynamics (PIMD) implemented in i-PI[12] package, together with our machine learning force field to evaluate the nuclear quantum effects (NQEs). For comparison, we run a PIMD simulation with 16 beads and a classical MD (CMD) for MgO_4H_6 at 5000 K, 400 GPa. We compare their mean square displacement (MSD) (a) and radial distribution function (RDF) between O-H, H-H and O-O (b)-(d).

Table. S1 | The thermal conductivities of the $Mg_2O_3H_2$ at 3000 K, 400 GPa with different cell size.

Size	Thermal conductivity ($Wm^{-1}K^{-1}$)
4x4x4	9.528
6x6x6	9.010
8x8x8	9.284

Table. S2 | The ratio of the thermal conductivities to the electrical conductivities in Mg-O-H system at 600 GPa in the pressure range from 3000 to 6000 K. Table (a-c) represent the results of the $Mg_2O_3H_2$, the MgO_3H_4 and the MgO_4H_6, respectively.

(a)	Thermal conductivity ($Wm^{-1}K^{-1}$)	Electrical conductivity (Ωcm^{-1})	Proton Diffusion rate ($10^{-9}m^2s^{-1}$)	κ/σ ($10^2W\Omega^{-1}K^{-1}$)
3000K	13.439	0.1982	0.3589	67.803
4000K	12.209	1.3919	3.3600	8.772
5000K	13.531	3.0365	9.1629	4.456
6000K	12.094	3.0555	11.0656	3.958

(b)	Thermal conductivity ($Wm^{-1}K^{-1}$)	Electrical conductivity (Ωcm^{-1})	Proton Diffusion rate ($10^{-9}m^2s^{-1}$)	κ/σ ($10^2W\Omega^{-1}K^{-1}$)
3000K	21.323	32.8287	26.2993	0.650
4000K	21.733	53.6493	57.3051	0.405
5000K	20.425	55.5741	74.2013	0.368
6000K	20.388	58.0070	92.9398	0.351

(c)	Thermal conductivity ($Wm^{-1}K^{-1}$)	Electrical conductivity (Ωcm^{-1})	Proton Diffusion rate ($10^{-9}m^2s^{-1}$)	κ/σ ($10^2W\Omega^{-1}K^{-1}$)
3000K	27.667	37.2953	25.8101	0.633
4000K	24.863	85.3944	78.7960	0.291
5000K	23.245	87.7378	101.1980	0.265
6000K	27.895	98.9170	136.9106	0.234

Structure parameters

CIF file of Pnma $Mg_2O_3H_2$ at 400 GPa:

```
data_1
_audit_creation_date                    2022-01-11
_symmetry_space_group_name_H-M       'PNMA'
_symmetry_Int_Tables_number           62
_symmetry_cell_setting                 orthorhombic
loop_
_symmetry_equiv_pos_as_xyz
   x,y,z
   -x+1/2,-y,z+1/2
   -x,y+1/2,-z
   x+1/2,-y+1/2,-z+1/2
   -x,-y,-z
   x+1/2,y,-z+1/2
   x,-y+1/2,z
   -x+1/2,y+1/2,z+1/2
_cell_length_a                          4.6498
_cell_length_b                          4.5257
_cell_length_c                          4.7986
_cell_angle_alpha                       90.0000
_cell_angle_beta                        90.0000
_cell_angle_gamma                        90.0000
loop_
_atom_site_label
_atom_site_type_symbol
_atom_site_fract_x
_atom_site_fract_y
_atom_site_fract_z
_atom_site_U_iso_or_equiv
_atom_site_adp_type
_atom_site_occupancy
O1       O      -0.34785     0.99675     0.34987     0.00000   Uiso     1.00
H1       H        0.13000     0.43165     0.55139     0.00000   Uiso     1.00
Mg1      Mg     -0.14292     0.25000     0.16394     0.00000   Uiso     1.00
Mg5      Mg     -0.15425     0.75000     0.14145     0.00000   Uiso     1.00
O5       O      -0.50210     0.75000     0.99971     0.00000   Uiso     1.00
```

CIF file of *C*222_1 MgO_3H_4 at 700 GPa:

```
data_2
_audit_creation_date                    2022-01-12
_symmetry_space_group_name_H-M       'C2221'
_symmetry_Int_Tables_number           20
_symmetry_cell_setting                 orthorhombic
```

```
loop_
_symmetry_equiv_pos_as_xyz
   x,y,z
   -x,-y,z+1/2
   -x,y,-z+1/2
   x,-y,-z
   x+1/2,y+1/2,z
   -x+1/2,-y+1/2,z+1/2
   -x+1/2,y+1/2,-z+1/2
   x+1/2,-y+1/2,-z
_cell_length_a                          2.1307
_cell_length_b                          6.7294
_cell_length_c                          5.2525
_cell_angle_alpha                       90.0000
_cell_angle_beta                        90.0000
_cell_angle_gamma                       90.0000
loop_
_atom_site_label
_atom_site_type_symbol
_atom_site_fract_x
_atom_site_fract_y
_atom_site_fract_z
_atom_site_U_iso_or_equiv
_atom_site_adp_type
_atom_site_occupancy
O2       O       0.99637    0.13944    1.44435    0.00000   Uiso    1.00
H1       H       0.63756    0.10205    0.54501    0.00000   Uiso    1.00
H3       H       0.32146    0.52431    1.14269    0.00000   Uiso    1.00
Mg1      Mg      0.00000    0.25111    0.75000    0.00000   Uiso    1.00
O1       O       0.50000    0.06173    0.75000    0.00000   Uiso    1.00
```

CIF file of $P4_2/n$ MgO_4H_6 at 300 GPa:

```
data_3
_audit_creation_date                    2022-01-12
_symmetry_space_group_name_H-M          'P 42/N'
_symmetry_Int_Tables_number             86
_symmetry_cell_setting                  tetragonal
loop_
_symmetry_equiv_pos_as_xyz
   x,y,z
   -x,-y,z
   -y+1/2,x+1/2,z+1/2
   y+1/2,-x+1/2,z+1/2
   -x+1/2,-y+1/2,-z+1/2
```

```
   x+1/2,y+1/2,-z+1/2
   y,-x,-z
   -y,x,-z
_cell_length_a                              5.3155
_cell_length_b                              5.3155
_cell_length_c                              2.2326
_cell_angle_alpha                          90.0000
_cell_angle_beta                           90.0000
_cell_angle_gamma                            90.0000
loop_
_atom_site_label
_atom_site_type_symbol
_atom_site_fract_x
_atom_site_fract_y
_atom_site_fract_z
_atom_site_U_iso_or_equiv
_atom_site_adp_type
_atom_site_occupancy
O1       O       0.23182    0.57764    0.98440    0.00000   Uiso    1.00
H1       H       0.64338    0.93230    1.27364    0.00000   Uiso    1.00
Mg1      Mg      0.50000    0.50000    0.50000    0.00000   Uiso    1.00
H5       H       0.25000    0.75000    1.25000    0.00000   Uiso    1.00
```

References

1. Hernandez, J. A. & Caracas, R. Superionic-Superionic Phase Transitions in Body-Centered Cubic H2O Ice. *Phys. Rev. Lett.* **117**, 1–5 (2016).
2. Cheng, B., Bethkenhagen, M., Pickard, C. J. & Hamel, S. Phase behaviours of superionic water at planetary conditions. *Nat. Phys.* **17**, 1228–1232 (2021).
3. Liu, C. *et al.* Multiple superionic states in helium–water compounds. *Nat. Phys.* **15**, 1065–1070 (2019).
4. Redmer, R., Mattsson, T. R., Nettelmann, N. & French, M. The phase diagram of water and the magnetic fields of Uranus and Neptune. *Icarus* **211**, 798–803 (2011).
5. Pickard, C. J., Martinez-Canales, M. & Needs, R. J. Decomposition and terapascal phases of water ice. *Phys. Rev. Lett.* **110**, 1–5 (2013).
6. Li, H.-F. *et al.* Ultrahigh-Pressure Magnesium Hydrosilicates as Reservoirs of Water in Early Earth. *Phys. Rev. Lett.* **128**, 035703 (2022).
7. Gao, H. *et al.* Superionic Silica-Water and Silica-Hydrogen Compounds in the Deep Interiors of Uranus and Neptune. *Phys. Rev. Lett.* **128**, 35702 (2022).
8. Stackhouse, S., Stixrude, L. & Karki, B. B. Thermal conductivity of periclase (MgO) from first principles. *Phys. Rev. Lett.* **104**, 1–4 (2010).
9. Tang, X. & Dong, J. Lattice thermal conductivity of MgO at conditions of Earth's interior. *Proc. Natl. Acad. Sci. U. S. A.* **107**, 4539–4543 (2010).
10. Imada, S. *et al.* Measurements of lattice thermal conductivity of MgO to core-mantle boundary pressures. *Geophys. Res. Lett.* **41**, 4542–4547 (2014).
11. Hofmeister, A. M. Thermal diffusivity and thermal conductivity of single-crystal MgO and Al2O3and related compounds as a function of temperature. *Phys. Chem. Miner.* **41**, 361–371 (2014).
12. Ceriotti, M., More, J. & Manolopoulos, D. E. I-PI: A Python interface for ab initio path integral molecular dynamics simulations. *Comput. Phys. Commun.* **185**, 1019–1026 (2014).
